# Radiologic Image of a Child with Leukemia Who Developed Sepsis and Fulminant Thrombosis during Induction Therapy

**DOI:** 10.4274/tjh.2015.0046

**Published:** 2016-02-17

**Authors:** Eda Ataseven, Ömer Özden, Şebnem Yılmaz Bengoa, Handan Güleryüz, Murat Duman, Hale Ören

**Affiliations:** 1 Dokuz Eylül University Faculty of Medicine, Department of Pediatric Hematology, İzmir, Turkey; 2 Dokuz Eylül University Faculty of Medicine, Department of Pediatric Intensive Care, İzmir, Turkey; 3 Dokuz Eylül University Faculty of Medicine, Department of Pediatric Radiology, İzmir, Turkey; 4 Dokuz Eylül University Faculty of Medicine, Department of Pediatric Emergency, İzmir, Turkey

**Keywords:** Acute leukemia, sepsis, Thrombosis

In a 5-year-old girl with acute lymphoblastic leukemia (ALL), febrile neutropenia occurred in the induction phase of chemotherapy. She was not using a central venous catheter. Despite empiric antibiotics, she developed tachypnea, bilateral rales, and disseminated intravascular coagulation (DIC). Viral, bacterial, and fungal investigations were unremarkable. Thorax and abdominal computed tomography showed bilateral consolidation areas in the lungs and multiple infarcts in the left lower lobe of the lungs, the liver, the spleen, the kidneys, and the intestines (Figure 1). Heparin infusion was started. No inherited prothrombotic defect could be shown; antiphospholipid antibodies were negative. She died of pulmonary failure.

Sepsis secondary to an unknown pathogen is the most common cause of mortality and the overall risk of symptomatic thrombosis is 5.2% in ALL [1,2,3]. Despite a high incidence of central nervous system and upper venous system events, widespread thromboembolism seems to be rare [3,4]. Our patient had multiple acquired risk factors, such as leukemia, concurrent administration of Escherichia coli asparaginase and prednisone, infection, and DIC. After administration of anticoagulant therapy, patients usually show improvement, but in our patient we could not reduce the occlusive events. This case is a good reminder for hematologists that the onset of neutropenic fever may be very aggressive and thrombotic events may occur rapidly and may be fulminant in children with ALL.

## Figures and Tables

**Figure 1:**
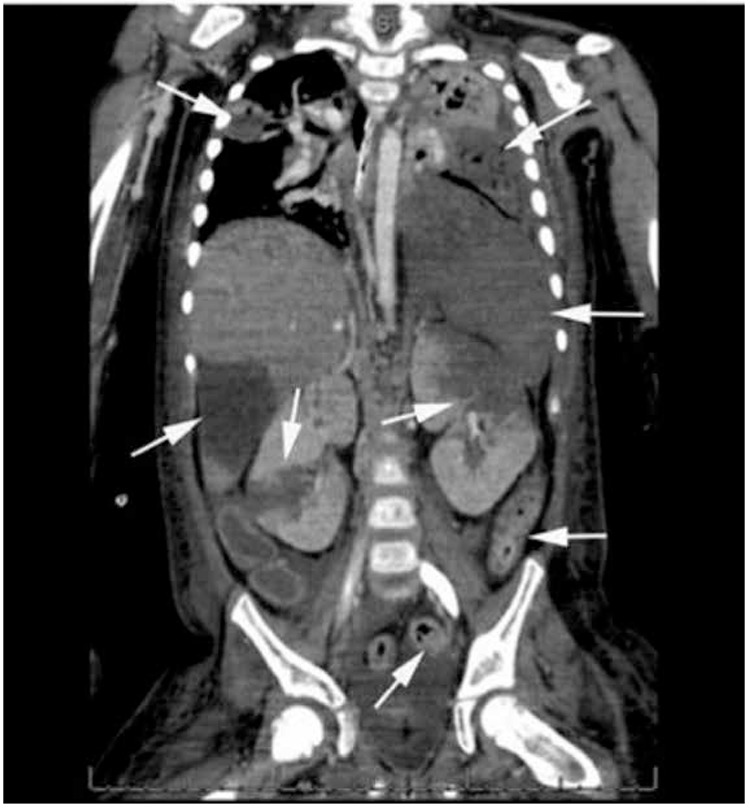
Thorax and abdominal computed tomography of the patient demonstrating bilateral areas of consolidation in the lung parenchyma and multiple infarcts in the left lower lobe of the lungs, in the liver, in the spleen, in the left upper lobe of the left and middle zone of the right kidney, and in some parts of the intestines (arrows).
